# Limb Ischemic Necrosis Secondary to Microvascular Thrombosis: A Brief Historical Review

**DOI:** 10.1055/s-0044-1786356

**Published:** 2024-04-30

**Authors:** Theodore E. Warkentin

**Affiliations:** 1 Department of Pathology and Molecular Medicine, McMaster University, Hamilton, Ontario, Canada; 2 Department of Medicine, McMaster University, Hamilton, Ontario, Canada; 3 Service of Benign Hematology, Hamilton Health Sciences, Hamilton General Hospital, Hamilton, Ontario, Canada

**Keywords:** acute ischemic hepatitis (“shock liver”), disseminated intravascular coagulation, heparin-induced thrombocytopenia, natural anticoagulants, symmetrical peripheral gangrene, venous limb gangrene

## Abstract

Ischemic limb injury can be broadly classified into arterial (absent pulses) and venous/microvascular (detectable pulses); the latter can be divided into two overlapping disorders—venous limb gangrene (VLG) and symmetrical peripheral gangrene (SPG). Both VLG and SPG feature predominant acral (distal) extremity ischemic necrosis, although in some instances, concomitant nonacral ischemia/skin necrosis occurs. Historically, for coagulopathic disorders with prominent nonacral ischemic necrosis, clinician-scientists implicated depletion of natural anticoagulants, especially involving the protein C (PC) system. This historical review traces the recognition of natural anticoagulant depletion as a key feature of nonacral ischemic syndromes, such as classic warfarin-induced skin necrosis, neonatal purpura fulminans (PF), and meningococcemia-associated PF. However, only after several decades was it recognized that natural anticoagulant depletion is also a key feature of predominantly acral ischemic microthrombosis syndromes—VLG and SPG—even when accompanying nonacral thrombosis is not present. These acquired acral limb ischemic syndromes typically involve the triad of (a) disseminated intravascular coagulation, (b) natural anticoagulant depletion, and (c) a localizing explanation for microthrombosis occurring in one or more limbs, either deep vein thrombosis (helping to explain VLG) or circulatory shock (helping to explain SPG). In most cases of VLG or SPG there are one or more events that exacerbate natural anticoagulant depletion, such as warfarin therapy (e.g., warfarin-associated VLG complicating heparin-induced thrombocytopenia or cancer hypercoagulability) or acute ischemic hepatitis (“shock liver”) as a proximate factor predisposing to severe depletion of hepatically synthesized natural anticoagulants (PC, antithrombin) in the setting of circulatory shock.

Microvascular necrosis as a complication of disseminated intravascular coagulation (DIC) is an important pathophysiological mechanism underlying acral (distal extremity) ischemic limb injury in two main clinical settings. One setting is acral ischemic limb injury in a patient with deep vein thrombosis (DVT), an entity known as “venous limb gangrene” (VLG). 1 Another is the clinical picture of multilimb acral ischemic limb injury occurring in patients with circulatory shock, an entity known as “symmetrical peripheral gangrene” (SPG). 1 DIC is an important feature seen in most patients with VLG or SPG. 1

However, VLG and SPG are not only associated with DIC, but also with oftentimes profound depletion of natural anticoagulant factors, such as protein C (PC), antithrombin (AT), and protein S (PS). In essence, acral limb ischemic injury due to microvascular thrombosis results from profoundly disturbed procoagulant–anticoagulant balance, with distinct localizing features: (a) proximal DVT predisposing to acral ischemic limb injury in the same limb with the DVT and (b) circulatory “shock” predisposing to largely symmetrical acral ischemic injury in SPG. 1

Thus, these two distinct (but overlapping) entities—VLG and SPG—share a common pathogenesis that feature the clinical triad of (a) procoagulant DIC, (b) concomitant natural anticoagulant depletion, and (c) a localizing predisposition (DVT and shock, respectively, for VLG and SPG); moreover, there is a (d) fourth characteristic feature, namely a characteristic time interval—usually 1.5 to 5 days (median, 3 d) between the initiation of the processes that lead to natural anticoagulant depletion (e.g., onset of underlying DIC trigger, administration of vitamin K antagonist [VKA] therapy, occurrence of “shock liver,” and so forth), and the onset of ischemic limb injury, thus presenting a characteristic clinical tetrad. Fig. 1 summarizes the conceptual triad and tetrad of VLG and SPG.

**Fig. 1:**
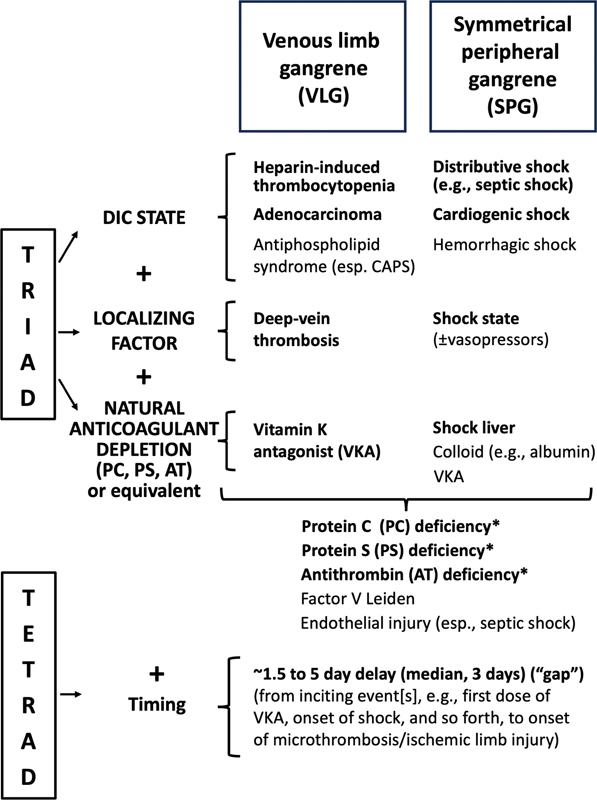
Triad and tetrad of venous limb gangrene and symmetrical peripheral gangrene syndromes. The characteristic “triad” of venous limb gangrene (VLG) and symmetrical peripheral gangrene (SPG) comprises a DIC state, localizing factor, and natural anticoagulant depletion; however, the underlying components of the triad differ between VLG and SPG. With the addition of the characteristic timing between the onset of one or more inciting events (e.g., first dose of warfarin, onset of circulatory shock and concomitant shock liver), also known as the “gap,” the four key clinical features can be considered a “tetrad.” **Bold text** indicates features that are more commonly encountered than features shown in regular (nonbold) text. For example, catastrophic antiphospholipid syndrome (CAPS) and hemorrhagic shock are less common explanations for VLG and SPG, respectively, than the other disorders listed in bold text (VLG: heparin-induced thrombocytopenia, adenocarcinoma; SPG: distributive [septic] shock, cardiogenic shock). * indicates that the deficiency of PC, PS, and AT is usually acquired; however, occasionally, there is underlying hereditable (congenital) deficiency of a natural anticoagulant. AT, antithrombin; DIC, disseminated intravascular coagulation; PC, protein C; PS, protein S; VKA, vitamin K antagonist.

This brief historical review traces the recognition of these acral limb ischemic syndromes, the identification of DIC and shock as relevant pathophysiological contributors, and the discovery (with their implication in limb ischemia pathogenesis) of the three natural anticoagulant factors, PC, PS, and AT. Based on the historical timeline, and my clinical experience through collegial discussions and review of case records, my thesis is that when patients develop prominent *nonacral* necrosis—such as typically seen in warfarin-associated skin necrosis or infection-associated purpura fulminans (PF)—the clinician-scientist is quick to invoke a disturbance in natural anticoagulants. In contrast, for predominantly *acral* limb ischemic syndromes (SPG, VLG), the clinician-scientist may be unaware of the pathophysiological role of natural anticoagulant depletion.

Table 1 lists key dates regarding the history of the understanding of acral ischemic limb injury. 2 3 4 5 6 7 8 9 10 11 12 13 14 15 16 17 18 19 20 21 22 23 24 25 26 27 28 29 30 31 32 33 34 35 36 37 38 39 40 41 42 43 44 45 46 47 48

**Table 1:** Historical timeline of ischemic limb injury secondary to venous/microvascular thrombosis

Year	Event	References
1859	Hueter reports case of venous limb gangrene	2
1884	Guelliot describes “infectious purpura fulminans” ( *Fr* “purpura infectieux foudroyant”)	3
1891	Hutchinson reported case of “severe symmetrical gangrene of the extremities” as permanent sequelae following nonfatal acute myocardial infarction	4
1924	Sanarelli (1924), Shwartzman (1928), and Apitz (1935) establish experimental animal models that became known as the localized and generalized Shwartzman phenomenon (early models of DIC)	5 6 7
1938	Gregoire uses the term “phlegmasia cerulea dolens” (severe venous limb ischemia)	8
1938	Fishberg uses the term “symmetrical peripheral gangrene” complicating heart failure for two patients (no clinical details provided)	9
1939	Perry and Davie report patient with SPG complicating terminal congestive heart failure; at postmortem, cardiac cirrhosis was also identified	10
1951	Schneider introduces the term “disseminated intravascular coagulation”	11
1954	Seegers et al identify antithrombin (AT; formerly, ATIII) as substance in plasma that inactivates thrombin (i.e., AT is the first natural anticoagulant discovered)	12
1954	Verhagen describes coumarin-associated skin necrosis	13
1958	First report implicating norepinephrine in contributing to SPG in myocardial infarction	14
1965	AT deficiency identified as the first hereditary hypercoagulable disorder	15 16
1972	Robboy et al show that microvascular thrombosis explains DIC-associated SPG	17 18
1976	(Re)discovery a of protein C (PC, formerly, called autoprothrombin II-A)	19 20 21 22
1979	DiScipio and Davie discover protein S	23
1981	Griffin et al identify PC deficiency as risk factor for hereditary thrombosis	24
1983	Branson et al implicate homozygous PC deficiency in neonatal purpura fulminans	25
1983	Broekmans et al implicate heterozygous PC deficiency in warfarin-induced necrosis	26
1984	Comp and Esmon identify PS deficiency as risk factor for hereditary venous thrombosis	27
1985	Molos and Hall identify DIC as “most common underlying condition” associated with SPG	28
1987	Acquired deficiencies in PC and PS are common in meningococcemia-associated PF	29
1990	Homozygous PS deficiency implicated as another explanation for neonatal PF	30 31
1990	*Seminars in Thrombosis and Hemostasis* publishes six comprehensive reviews on PF (congenital, acquired), warfarin-induced skin necrosis, and the Shwartzman reaction	32 33 34 35 36 37
1995	Levin et al report acquired PS deficiency in childhood post-varicella PF	38
1997	Warkentin et al report warfarin-associated VLG (acral usually without nonacral necrosis) complicating HIT; HIT-associated DIC plus acquired PC depletion is posited	39
2000	Knight et al emphasize association between SPG and shock: SPG occurs in “patients who are septic and have DIC and in nonseptic patients who have cardiogenic … shock”	40
2001	Taylor et al (Marcel Levi, senior author) propose DIC scoring system	41
2012	Siegal et al report patient with SPG and proximate shock liver (called “acute hepatic necrosis”) and suggest impaired hepatic synthesis of natural anticoagulants (PC, AT) is relevant to SPG pathogenesis	42
2015	Warkentin notes in *Seminars in Thrombosis and Hemostasis* that SPG features high frequency of DIC (100%), lactic acidemia (100%), and proximate shock liver (>90%)	43
2015	Warkentin et al report warfarin-associated venous limb gangrene (acral limb necrosis with minimal central skin necrosis) in setting of adenocarcinoma-associated DIC	44
2018	Warkentin (2018) and Levy et al (2019) challenge concept that vasopressor therapy is the major explanation for SPG (rather, DIC + shock + natural anticoagulant depletion)	45 46
2020	Warkentin et al suggest colloids (albumin, high-dose IVIG) could predispose to SPG	47
2021	Warkentin and Ning review clinical features and pathogenesis of SPG in critical illness	48

Abbreviations: AT, antithrombin; DIC, disseminated intravascular coagulation; HIT, heparin-induced thrombocytopenia; IVIG, intravenous immunoglobulin; PC, protein C; PF, purpura fulminans; PS, protein S; SPG, symmetrical peripheral gangrene; VLG, venous limb gangrene.

a Protein C listed as “rediscovery” in 1976 by Stenflo, 19 as it became evident 20 that protein C was identical to “autoprothrombin II-A” discovered in 1960 21 (as recounted by Owen 22 ).

## Natural Anticoagulants

Table 2 lists properties of the three natural anticoagulant factors, PC, PS, and AT. 49 50 One noteworthy fact is that PC and PS are present in very low molar concentrations—approximately 70 and 250 nM, respectively, which are much lower (∼5,000–20,000 × ) than the procoagulant factor, prothrombin (1,400 nM), although relatively similar to the concentrations typically observed for zymogen factor X (135–170 μM). Moreover, the half-life of PC (∼6–8 h) is also much lower than all of the other procoagulant factors (e.g., prothrombin, 60 h; factor XI, 60 h; factor X, 40 h; factor V, 12 h), except for factor VII (3–6 h). 1 42 These features make PC especially vulnerable to depletion during consumptive coagulopathic states such as DIC, 51 especially if there is concomitant impaired factor production (coumarin treatment, liver dysfunction). Another consideration is that the half-life of PC becomes even shorter (2–3 h) in consumptive coagulopathy. 52

**Table 2:** Three circulating natural anticoagulant proteins

Anticoagulant protein	Features (bullet points)
Protein C (other names: autoprothrombin IIA; factor XIV)	• Glycoprotein (∼23% carbohydrate) circulates as a disulfide bond-linked two-chain 419-amino acid-polypeptide (62 kDa) • Vitamin K-dependent (GLA domain containing 9 gla residues) • Concentration, 4 to 6 μg/mL (∼65–95 nM), reported as reference range approximately 0.65–1.35 U/mL (functional activity, SI system) • Half-life, 6 to 8 hours (shortened in DIC states) • Synthesis: liver • Serine protease that circulates as inactive zymogen; when activated by thrombin (only when thrombin is bound to thrombomodulin, facilitated by endothelial protein C receptor), activated protein C degrades factors Va and VIIIa through proteolytic cleavage; potentiates fibrinolysis by neutralization of plasminogen activator inhibitor-I
Protein S	• Glycoprotein (∼8% carbohydrate) circulates as a 635-amino acid-polypeptide (70 kDa) • Vitamin K-dependent (GLA domain contains 10 gla residues) • Concentration, 17 to 25 μg/mL (∼240–350 nM), 40% free and 60% bound to C4b-binding protein; only free PS (FPS) has anticoagulant function (FPS reference range, males, ∼0.74–1.46 U/mL; females, ∼0.55–1.24 U/mL [antigen assays]) • Half-life, 30 hours • Synthesis: liver (predominant), but also endothelial cells, Leydig cells (testis), and megakaryocytes • Regulatory protein (not a serine protease) that serves as a cofactor for the anticoagulants, activated protein C and tissue factor pathway inhibitor; PS also directly inhibits factors VIIIa (within tenase complex) and Va and Xa (within prothrombinase complex)
Antithrombin	• Glycoprotein (∼15% carbohydrate) circulates as a single-chain 432-amino acid-polypeptide (58 kDa) • Concentration, 125 to 160 μg/mL (∼2.5 mM), reported either as reference range ∼0.80–1.20 U/mL (functional activity, SI system) or in percentage values (reference range, ∼80–120% activity level) • Half-life, approximately 65 hours • Synthesis: liver • Primary inhibitor of several activated serine proteases, for example, thrombin (IIa), IXa, Xa, XIa, XIIa, kallikrein, plasmin, urokinase, trypsin; AT forms 1:1 irreversible complex with target enzyme • AT binding to thrombin (IIa) is accelerated approximately 1,000× by heparin

Abbreviations: AT, antithrombin; DIC, disseminated intravascular coagulation; PC, protein C; PS, protein S.

References for this Table. 49 50 Natural anticoagulant reference ranges shown are usual adult ranges. Concentrations given in U/mL are synonymous with the designation IU/mL (per the SI system). Whereas PC and AT levels are usually measured using functional (activity) assays, PS levels are usually measured using antigen assays.

## Natural Anticoagulant Deficiency in Nonacral Microvascular Thrombosis

### Neonatal Purpura Fulminans

It is well-established that profound deficiency of a natural anticoagulant can cause dramatic microvascular thrombosis. One example is hereditary absence of PC, a condition that results in neonatal PF; both homozygous and compound heterozygous mutations are reported. 25 52 53 54 55 56 57 58 59 Complete absence of PS can also cause neonatal PF. 30 31

Affected patients develop multiple nonacral and acral areas of skin necrosis, approximately 12 to 24 hours following birth (some neonates also develop in utero cerebral and ophthalmic thrombotic complications). Interestingly, a picture of DIC develops (thrombocytopenia, hypofibrinogenemia); during PC replacement studies, it was shown that DIC markers are evident when PC activity levels fall below 10 to 25% of normal. 52 Treatment with frozen plasma (FP) prevents skin lesions and leads to resolution of DIC (although recurrence will occur if FP infusion is not continued at regular intervals); PC concentrates are also effective for PC deficiency-related PF, 52 including when administered by subcutaneous injection. 59 Treatment with coumarin anticoagulation can permit discontinuation of FP without recurrence of skin lesions in some patients. 56 58

Interestingly, no disorder of congenital absence of AT has been reported, presumably due to embryonic lethality.

### Infection-associated Purpura Fulminans

Many bacteria—especially the encapsulated organisms, meningococcus ( *Neisseria meningitidis* ), pneumococcus ( *Streptococcus pneumoniae* ), *Haemophilus influenzae* —can trigger DIC and PF in some patients, especially postsplenectomy, with functional asplenism, or having other immunological abnormalities (e.g., properdin deficiency). 1 Other implicated pathogens include *Streptococcus pyogenes* (and other streptococcal species), *Staphylococcus* species, *Escherichia coli* , and Capnocytophaga species (associated with dog or human bites), among others. Nonbacterial triggers (rickettsia, malaria, certain viruses) are also reported. 1

Historically, a role for natural anticoagulant depletion in PF was first shown for meningococcemia. Powars and colleagues 29 showed reduced PC and PS activity in six children with meningococcemia-associated PF, providing a rationale for PC replacement treatment. 60 PC activation impairment is likely also related to endothelial injury, with downregulation of the thrombomodulin (TM)-endothelial PC receptor pathways. 61 Intriguingly, a case–control study found that factor V Leiden (a mutation that impairs factor Va proteolysis by activated PC) was associated with a three-fold increase in risk for PF (21 vs. 7%) in meningococcemia. 62

Lerolle and colleagues 63 studied 20 patients with PF (meningococcus, *n* = 2; pneumococcus, *n* = 4; other *Streptococcus* , *n* = 5; *Staphylococcus aureus* , *n* = 2; gram-negative bacilli, *n* = 3). Controls included severe sepsis patients (with [ *n* = 15] and without [ *n* = 20] DIC) but without PF. Patients with PF (vs. controls) had higher Sequential Organ Failure Assessment scores (indicating greater organ dysfunction) and higher mortality rate (80 vs. 46%). Interestingly, the authors remarked that “the maximum catecholamine infusion rate was not different between the groups.” 63 Histopathology showed microthrombosis in PF patients (but not controls), with decrease in TM and endothelial PC receptor expression, but increase in plasminogen-activating inhibitor-1 expression. Compared with controls, PF patients had lower platelet counts, higher D -dimer levels, but similar fibrinogen levels; however, significantly lower levels of all three natural anticoagulants—PC, PS, and AT—were seen in PF patients versus controls.

### Postinfectious Purpura Fulminans

Certain infections are known to be subsequently complicated by PF even after the primary illness has resolved. The best-studied example is varicella-induced PS deficiency, a disorder of children (median age, ∼5 years), which appears to reflect a postviral autoantibody-mediated clearance of PS that occurs approximately 7 to 10 days post-varicella or herpesvirus 6 (HHV-6) infection 38 64 ; some patients have large vessel thromboembolism rather than the picture of small vessel PF. 65 Interestingly, secondary occurrence of DIC appears to be an adverse risk factor, given that thrombocytopenia severity and low AT levels are associated with worse outcomes (e.g., amputation).

## Coumarin (Warfarin)-induced Skin Necrosis

The coumarin class of VKAs is a well-established cause of predominantly nonacral skin necrosis, known as “coumarin-induced skin necrosis” (CISN) and “warfarin-induced skin necrosis” (WISN). 13 26 66 Only a minority (∼10%) of patients with CISN/WISN have acral involvement. 66 The majority of patients appear to have hereditary abnormalities of the PC anticoagulant pathway, for instance, heterozygous PC deficiency, factor V Leiden, and so forth. In most patients with CISN, there is an underlying hypercoagulability state; accordingly, pathogenesis of CISN is believed to reflect disturbed procoagulant–anticoagulant balance. 66

## Disseminated Intravascular Coagulation is Triggered by Severe Protein C or Protein S Depletion

Patients with very low levels of PC (neonatal PF) or PS (postinfectious PF) typically develop thrombocytopenia, which (for PC deficiency) responds to PC replacement; this suggests that profound depletion of PC can result in DIC. 52 This finding is distinct from most of the other entities discussed in this review, where patients have an initial clear trigger for DIC, for example, heparin-induced thrombocytopenia (HIT), adenocarcinoma, septic shock, cardiogenic shock.

## Venous Limb Gangrene

It is surprising to many clinicians that limb ischemic necrosis can result from thrombosis that only involves veins. Although reports on venous gangrene date from over 150 years ago (e.g., Hueter 2 ), the concepts of venous gangrene, and the corresponding prodromal entity of “phlegmasia cerulea dolens” (PCD; swollen, cyanotic, painful [limb]), 8 was popularized by a 1907-born vascular surgeon, Henry Haimovici, who (unable to accept a prestigious position in Marseilles due to the outbreak of World War II) became chief of Vascular Surgery Montefiore Medical Center in New York City in 1945. 67 Haimovici wrote several articles on VLG and PCD, 68 69 and summarized all reported cases to date in his 1971 book, *Ischemic Forms of Venous Thrombosis* . 70

Among the concepts espoused by Haimovici were reversibility of PCD (but not VLG), and presence of pulses in PCD and VLG (although sometimes impalpable or transiently nondetectable); approximately 25% of patients with VLG had underlying cancer. 69 Most often, one limb was affected, although in approximately 20% of cases, both lower limbs were affected.

Some of the cases listed by Haimiovici are consistent with coumarin-associated microthrombosis. For example, one case highlighted in two publications 68 71 involved a 62-year-old male patient who developed left lower limb DVT 11 days after surgery for bladder neoplasm. Dicumarol, 100 mg first dose (i.e., 10–50 times the usual maintenance dose of 2–10 mg/d 72 ) was given, as well as papaverine. Four days later, the patient's distal left foot became painful, cold, and mottled, despite pulpable pedal pulses, progressing to gangrene of several toes and especially the plantar region; over the next several weeks, spontaneous amputation of several distal toes occurred. In my opinion, this case plausibly represents coumarin-associated VLG (discussed subsequently).

## Heparin-induced Thrombocytopenia and Coumarin-associated Venous Limb Gangrene

Ischemic limb injury secondary to arterial thrombosis was the earliest recognized complication of HIT, an adverse drug reaction caused by platelet-activating antiplatelet factor 4/heparin antibodies (for historical review: Warkentin 2018 73 ). However, some early cases of HIT-associated ischemic limb injury involved veins rather than arteries; in a 1979 paper by Towne and colleagues 74 who coined the term, “white clot syndrome” (platelet-rich arterial thromboemboli), two of the seven patients reported had “phlegmasia cerulea dolens of the lower extremity…that progressed to venous gangrene.”

In December 1992, while I attended the American Society of Hematology (ASH) meeting in Anaheim, CA, Professor John Kelton was called to see a patient at my hospital with HIT-associated DVT being managed by ancrod (defibrinogenating snake venom) and warfarin. Despite a supratherapeutic international normalized ratio (INR), the patient's limb was becoming progressively ischemic; Kelton noted a patch of skin necrosis on the abdominal wall, suggestive of WISN. The patient ultimately required limb amputation, and the pathology showed thrombosis involving large and small veins, as well as the microvasculature (venules). After returning from the ASH meeting, I wondered whether warfarin might also have been responsible for the microthrombosis associated with acral ischemic limb loss. A second patient 2 months later—who developed severe phlegmasia in a HIT-associated upper limb DVT, also managed with warfarin and ancrod—further supported this concept.

Our hypothesis was tested with blood samples from these two index patients, as well as two other patients with VLG in whom plasma samples were available. We also had 67 plasma samples from 12 patients with HIT treated with warfarin who did not develop VLG (controls). We found evidence for a severe disturbance in procoagulant–anticoagulant balance, that is, the samples from the four patients with VLG had high ratios of thrombin–antithrombin complex (a marker of thrombin generation) to PC activity, compared with the controls. 39 In total, we identified 8 patients with VLG, and 10 with arterial thrombosis complicating HIT. We found supratherapeutic INR levels in the patients with VLG versus controls (5.8 vs. 3.1; *p* < 0.001). Ironically, because patients with limb artery thrombosis usually had their limbs salvaged through arterial thromboembolectomies, the occurrence of limb amputation was greater in the patients with VLG than with HIT-associated limb artery thrombosis (6 vs. 3 patients).

Our finding that warfarin can cause VLG in the setting of HIT has been confirmed by others. 75 76 These observations helped change clinical practice: warfarin is now considered contraindicated for management of the acute phase of HIT. 77

## Heparin-induced Thrombocytopenia-associated Venous Limb Gangrene without Proximate Warfarin

In my experience, warfarin treatment is implicated in the majority (>90%) of patients with HIT-associated VLG. However, the exceptions generally involve unusually severe HIT, including patients with overt DIC. One example is a patient reported in *Seminars of Thrombosis and Hemostasis* , 43 who had a platelet count nadir of only 10 × 10 ^9^ /L, along with overt DIC (peak INR, 1.9; fibrinogen nadir, 1.0 g/L; D -dimer levels >20 μg/mL fibrinogen equivalent units). Interestingly, this patient also had acquired natural anticoagulant depletion, with a nadir AT level of only 0.39 U/mL (reference range, 0.77–1.25 U/mL) and a nadir PC activity level of only 0.48 U/mL (reference range, 0.70–1.80 U/mL).

## Adenocarcinoma and Coumarin-associated Venous Limb Gangrene

Anecdotal reports by myself 78 and others 76 79 80 indicated that VLG could also complicate coumarin treatment for some patients with cancer. In 2015, my colleagues and I reported a case-series of 10 patients with warfarin-associated VLG complicating DVT in the setting of adenocarcinoma-associated DIC. 44 We observed a characteristic clinical profile: patients typically presented as “idiopathic” DVT (i.e., underlying cancer had not yet been identified in most patients), with an increase in platelet counts during the initial phase of heparin treatment (reflecting heparin control of underlying cancer-associated DIC), followed by an abrupt decrease in platelet count upon stopping heparin (once a therapeutic level of anticoagulation was achieved by warfarin), and abrupt worsening of lower limb ischemia (phlegmasia) with a concomitant increase in the INR to supratherapeutic levels (generally, >3.5), with ultimate progression to VLG. As in our preceding paper on HIT-associated VLG, 39 the available blood samples supported the concept of disturbed procoagulant–anticoagulant balance. 44

Although these 1997 and 2015 papers clearly implicated warfarin in the pathogenesis of VLG, this association was highly counterintuitive. Indeed, Haimovici himself—writing 10 years before our 1997 HIT-VLG paper—argued that Coumadin did not explain VLG. 81

## Symmetrical Peripheral Gangrene

SPG usually represents microvascular thrombosis leading to largely symmetrical losses of distal extremities, most often in the setting of circulatory shock and DIC; the typical clinical picture is that of acral limb ischemia despite palpable or Doppler-identifiable arterial pulses during the progression to tissue necrosis, in the setting of critical illness. 1 Although a minority of SPG occurs in non-DIC settings, such as frostbite, inflammatory bowel disease, various hematological malignancies, and so forth, these are usually readily discernable by their occurrence in nonintensive care unit settings and otherwise distinct clinical profiles. 1 The first published case of SPG dates from 1891, 4 but the available information does not permit a clear determination of the underlying explanation.

In this review, I am considering SPG as distinct from PF, an entity which also features microvascular thrombosis, but generally with a prominent picture of multiple nonacral skin necrosis lesions. There is considerable overlap, however, as many patients with PF also have acral tissue necrosis, with risk for limb ischemic necrosis. However, whereas PF in the setting of DIC is widely recognized as representing failure of natural anticoagulant mechanisms, most clinicians seem not to be aware that natural anticoagulant depletion is also a hallmark of SPG.

## Symmetrical Peripheral Gangrene in Cardiology

SPG was reported in 1939 in the setting of heart failure. 10 Other cardiac disorders occasionally complicated by SPG included paroxysmal tachycardia, 82 congenital heart disease, 83 and acute myocardial infarction (AMI). 84 85 86 In some of these reports, however, preceding anticoagulant treatment for AMI 84 86 suggest the possibility of VKA treatment having caused or exacerbated SPG. Postmortem examination of a fatal case of AMI complicated by SPG showed centrilobular hepatic necrosis, 85 in keeping with a role of shock liver in SPG pathogenesis (discussed subsequently). One report of AMI-associated SPG 86 depicts well the typical time delay between onset of shock and subsequent occurrence of SPG:

“Except for a short interval of 2 hours, the blood pressure was unobtainable for a total of over 40 hours. … On the morning of the 4th hospital day, some 80 hours after the onset of shock, it was first noted that the tip of the nose, fingers and toes were very cyanotic. The cyanotic areas slowly spread, and by the 7th day had progressed to superficial gangrene symmetrically involving the toes, soles and heels, fingers, and end of the nose.”

The underlying theme of these reports is that SPG is a complication of cardiogenic shock. In 1952, Selzer reported occurrence of hypotension/shock in 69/528 (13%) of AMI patients 87 ; in his paper, one patient with SPG was described as follows:

“The patient persisted in the shocklike state for eight days. On the sixth day early gangrene of the fingertips and some discoloration of the tip of the nose was noted. Autopsy revealed an old myocardial infarction in the posterior wall of the left ventricle, a total occlusion of the right coronary artery by a thrombus, and massive myocardial infarction of the anterior wall of the left ventricle and the septum.”

None of these papers included any mention of platelet counts (not routinely performed prior to the 1970s); indeed, these reports preceded the modern concept of DIC. To illustrate, a 1965 case of SPG 88 complicating placement of mechanical mitral valve featured profound postoperative hypotension, onset of limb ischemia on postoperative day 4, with four-limb ischemic necrosis. Early liver dysfunction was shown by jaundice and elevated hepatic transaminase levels. Autopsy revealed left atrial mural thrombus, with evidence of infarction involving kidneys, brain, lung, liver, spleen. To a modern reader, various plausible diagnoses—for example, cardiogenic shock/DIC/shock liver with microthrombosis, HIT with coumarin-associated microthrombosis—were unknown six decades ago. Moreover, the paradox of a supratherapeutic INR as a marker of coumarin microthrombosis (a concept unknown in 1965) was suggested by the following statement: “Some difficulty was experienced in regulating the Coumadin dosage in the early postoperative period, possibly due to the compromised hepatic function, but the error was in the direction of too much drug rather than too little.” 88

## Symmetrical Peripheral Gangrene in Sepsis: Recognition of Disseminated Intravascular Coagulation and Shock

SPG as a complication of sepsis has been known for many years, both in association with PF (e.g., meningococcemia), but also distinct from PF. Guelliot 3 used the term “infectious purpura fulminans,” which contrasts with “purpura hemorrhagica fulminans,” 89 the latter consistent with a usually self-limited prohemorrhagic disorder such as acute childhood immune thrombocytopenia. In my review, SPG refers to a disorder with exclusive or predominant acral necrosis.

Molos and Hall reviewed SPG in 1985, 28 entitling their paper, “Symmetrical peripheral gangrene and disseminated intravascular coagulation.” This paper accomplished three tasks. First, they reported three new cases of SPG (one associated with *E. coli* sepsis) and reviewed 68 previously reported examples of SPG identified in the English-language literature. Second, their use of the term “SPG” in a review paper helped to create wider acceptance of this term—which hitherto had been used in some individual case reports. 90 91 92 93 94 Third, they emphasized an important connection, stating that “DIC” was a common feature among the cases. They opined: “Review of the medical literature shows a high association between SPG and DIC. SPG should therefore be considered a cutaneous marker of DIC.” 28

Their work followed that of Robboy, a pathologist working with the hematologist, Robert Colman (Editor of Colman's Textbook of *Hemostasis and Thrombosis* ). Robboy and colleagues' investigations also helped to link the characteristic symmetrical dermal abnormalities with the entity of DIC. 17 18 In their 1973 paper in British Journal of Dermatology, they wrote 18 :

“The acral cyanosis of DIC is gun-metal grey, sharply demarcated, and symmetrical, and the tips of the digits or nose may be blue-black, suggesting impending gangrene. Pulsations in arteries are intact (except when arterial catheters are present), which rules out embolic lesions; the capillary perfusion is grossly decreased, even when the skin is warm, reflecting the occlusive nature of the fibrin thrombi in small vessels. These characteristics plus the clinical setting of sepsis, shock, or neoplasia, make the diagnosis of the acral cyanosis in the more indolent diseases, Raynaud's phenomenon, chilblains, or acrocyanosis, unlikely.”

A consequential report by Knight et al 40 reaffirmed the role of DIC in the SPG pathogenesis, but these authors also emphasized the role of concomitant shock. Specifically, they wrote: SPG “occurs in patients who are septic and have disseminated intravascular coagulation and in nonseptic patients who have cardiogenic or hypovolemic shock.” They speculated that for SPG to occur, “there must be a low-flow state in the microcirculation of the affected parts.” Moreover, the authors noted that even among patients with DIC and shock, the occurrence of SPG was rare, causing the authors to speculate that there must be missing factors, such as “vasospasm either alone or in combination with pre-existing pathology in the microcirculation, sludging of platelet or fibrin degradation products in the microcirculation, the colder state of the distal parts relative to the trunk, or immunologic or molecular events not yet defined.” Indeed, as discussed in the next sections, “immunologic” (immunothrombosis) and “molecular events” (natural anticoagulant depletion) are critical factors in SPG pathogenesis.

## Natural Anticoagulant Depletion in Symmetrical Peripheral Gangrene

As noted earlier in this paper, acute infection complicated by DIC, shock, and PF is widely recognized to involve acquired depletion of natural anticoagulants. 29 60 63 However, in my experience, when there is exclusive or predominant acral ischemic necrosis, clinicians do not typically invoke natural anticoagulant depletion. Yet, severe depletion of natural anticoagulants is characteristic of these patients.

In 2012, Siegal, Cook, and I published a case of SPG complicating cardiogenic shock (the patient was not receiving vasopressors). 42 The patient had DIC and proximate shock liver. We proposed that *acute* liver dysfunction—with resulting impaired production of hepatically synthesized natural anticoagulants (AT, PC, PS)—could be a key factor in explaining SPG in patients with shock and DIC. Subsequently, I reported in 2015 in *Seminars of Thrombosis and Hemostasis* that in my experience >90% of patients with SPG in the setting of shock and DIC have proximate shock liver. 43 In support of this concept, the role of *chronic* liver disease in explaining thrombotic events via depletion of natural anticoagulants is well-established in the literature. 95 96

**Fig. 2:**
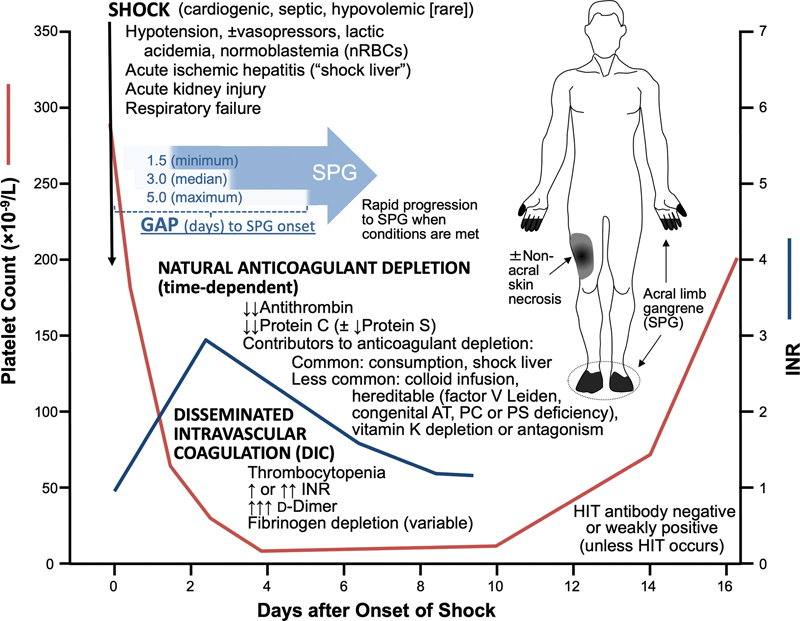
Schematic depiction of symmetrical peripheral gangrene (SPG) in critical illness. The clinical triad of ( **a** ) shock, ( **b** ) natural anticoagulant depletion, and ( **c** ) disseminated intravascular coagulation (DIC) is shown, along with the characteristic ( **d** ) temporal gap between onset of shock/DIC and onset of SPG, which reflects time-dependent decrease in natural anticoagulants to critically low levels. The gap is typically between 1.5 to 5 days (median, 3 d). A minority of patients with SPG will have nonacral skin necrosis. AT, antithrombin; HIT, heparin-induced thrombocytopenia; INR, international normalized ratio; nRBC, nucleated red blood cell; PC, protein C; PS, protein S.

Table 3 summarizes some of the key data from this 2012 case, 42 as well as other SPG cases I have reported, 1 47 48 97 98 99 consistent with the triad of shock (lactic acidemia), DIC (severe thrombocytopenia, elevated INR, greatly elevated D -dimer levels), and natural anticoagulant depletion, particularly of AT and PC. Fig. 2 summarizes the clinical and laboratory picture of SPG in critical illness.

**Table 3:** Markers of disseminated intravascular coagulation, shock, shock liver, and natural anticoagulant depletion in symmetrical peripheral gangrene: 10 cases reported by the author

Patient (age [yr], sex)	Shock (CS, SS)	Platelet count nadir ×10 ^9^ /L (150–400)	nRBCs per 100 WBC (0)	Lactate mmol/L (0.5–2.2)	ALT U/L (<40)	INR (0.8– 1.2)	Fibrinogen g/L (1.6–4.2)	D-Dimer μg/L (<500)	Antithrombin activity, U/mL (0.77–1.25)	Protein C activity, U/mL (0.70–1.80)	Free protein S antigen, U/mL (0.62– 1.44) a	References
62F	CS	15	87	17.6	2,468	6.0	0.7	44,600	0.20	<0.05	0.60	42
67F	CS	39	82	Acidemia	1,200	>11 b	0.7	>6,000	0.33	ND	ND	97
66F	CS	8	37	4.4	5,050	3.9	3.5	ND	0.46 c	0.58 c	ND	97
23F	CS/SS	17	11	15.0	2,280	3.0	1.1	26,920	0.32	<0.05	0.47	1
45F	SS	3	3	13.4	152 d	1.3	4.8	>20,000	0.24	0.22	0.28	98
61M	CS	37	5	8.3	1,168	2.7	0.3	9,950	0.35	0.17	0.79	99
45F	SS	4	2	8.8	29 e	1.7	3.1	>20,000	0.22	0.13	0.18	47
38F	SS	32	5	9.1	44 e	2.4	4.3	12,760	0.15	0.13	0.28	47
33F	CS	5	2	26.1	1,759	1.8	2.1	>20,000	0.30	0.25	0.56	48
74M	CS	17	7	4.0	358 e	1.6	2.5	16,000	0.52	0.29	0.91	48
**Median**												
53	NA	16	6	9.1	1,184	2.6	2.3	>20,000	0.31	0.17	0.52	–
**Interquartile range**												
40, 65	NA	6, 28	4, 31	8.3, 15.0	205, 2,150	1.7, 3.7	0.8, 3.4	12,760, >20,000	0.23, 0.35	0.13, 0.25	0.28, 0.65	–
**Range**												
23, 74	NA	3, 39	2, 87	4.0, 26.1	29, 5,050	1.3, >11	0.3, 4.8	9,950, 44,600	0.15, 0.52	<0.05, 0.58	0.18, 0.91	–

Abbreviations: ALT, alanine transaminase; CS, cardiogenic shock; INR, international normalized ratio; NA, not applicable; ND, not done; nRBC, nucleated red blood cell; SS, septic shock; WBC, white blood cell count; yr, years.

For all parameters, the data shown are either peak values (nRBCs, lactate, ALT, INR, D -dimer) or nadir values (platelet count, fibrinogen, antithrombin [AT] activity, protein C (PC) activity, free protein S antigen). Reference ranges shown are per my institution (Hamilton Health Sciences, Hamilton General Site).

a Reference range shown is for female patients; for male patients, the reference range is 0.78 to 1.61 U/mL.

b Patient was receiving argatroban at time INR measured >11.

c AT and PC activity were measured a few days after onset of symmetrical peripheral gangrene [SPG]; hence, values indicated are likely higher than the true nadir AT and PC activity levels, had they been measured at time of onset of SPG.

d Patient had chronic liver disease, potentially explaining role of hepatic dysfunction in the absence of marked transaminitis.

e Patients were receiving colloids (e.g., albumin) at time of onset of SPG, potentially implicating dilutional decrease in natural anticoagulants (in absence of marked transaminitis).

## Treatment of Incipient Symmetrical Peripheral Gangrene

Treatment of SPG—a topic beyond the scope of this review—is uncertain. In the future, growing clinician awareness of the characteristic clinical triad (shock, DIC, transaminitis) might in some circumstances offer the potential to prevent ischemic limb injury, if effective treatment is given during the characteristic temporal gap that constitutes the SPG tetrad ( Fig. 1 ). However, what constitutes “effective” treatment is unclear. In theory, anticoagulation with unfractionated heparin—the agent approved by the Food and Drug Administration to treat DIC 48 —and replacement of depleted natural anticoagulants might benefit some patients. However, heparin can predispose to bleeding in coagulopathic, critically ill patients, and it is not known whether heparin is even effective in preventing microvascular thrombosis in this clinical setting; moreover, thrombocytopenia and limb ischemia can raise the issue of immune HIT, 43 posing a (relative) contraindication to heparin, and direct thrombin inhibitors (e.g., argatroban, bivalirudin) could worsen SPG risk through inhibition of thrombin-mediated activation of PC. 100 Laboratory measurement of natural anticoagulant levels is not usually available quickly, and most blood banks do not have AT and PC concentrates available; PS concentrates do not exist. In my hospital (a cardiac surgery center), AT concentrates are usually available, and I have sometimes recommended their use when AT deficiency is proven or suspected. In contrast, PC concentrates are not usually timely available (due to requirement for patient-specific product approval and time to ship product), and I have therefore sometimes recommended use of FP in an attempt to increase natural anticoagulant levels. An important area of uncertainty is the use of specialized multifactor concentrates. For example, it is clear that 3-factor prothrombin complex concentrates (PCCs) are not appropriate for managing SPG, as these only contain *procoagulant* vitamin K-dependent factors (II, IX, and X, with minimal levels of factor VII) 101 ; in contrast, 4-factor PCCs contain roughly similar concentrations of the four procoagulant vitamin K-dependent factors (II, VII, IX, and X), but also contain the two vitamin K-dependent natural *anticoagulants* , PC and PS. 102 103 However, whether administration of such concentrated 4-factor (really, 6-factor) PCCs would provide net benefit or harm in preventing or ameliorating SPG is uncertain, although their use in infectious PF (meningococcemia) has been advocated (if PC concentrates are not available). 104 Intravenous vitamin K (given slowly over 30–60 min) is appropriate in a critically ill patient, as vitamin K deficiency—if it occurs—can exacerbate failure of the PC natural anticoagulant pathway. Transfusion of platelets and cryoprecipitate/fibrinogen concentrates might in theory promote microthrombosis in some situations, but the decision to withhold transfusion can be difficult (and potentially catastrophic) when the critically ill patient has severe thrombocytopenia and/or hypofibrinogenemia, and life-threatening (even fatal) bleeding poses significant risk. Further illustrating clinical uncertainty, it is possible that colloids such as albumin (often given to enhance intravascular volume) could contribute to microthrombosis secondary to natural anticoagulant depletion (through dilution), 47 giving another reason to consider FP if colloid administration is desired. Finally, it is my experience that once the conditions for microthrombosis are present, there is rapid evolution of this process with inevitability of SPG (even if the characteristic physical changes of tissue necrosis only become evident over the subsequent days); thus, once tissue injury is clinically apparent, it is likely too late to change the trajectory.

## Conclusion

Future work on SPG pathogenesis should focus on pathophysiology, including the timing and severity of natural anticoagulant depletion (implications for anticoagulant replacement), as well as other potential players, for example, immunothrombosis markers such as NETosis and endothelial injury. The development of relevant animal models could be helpful. I am aware of only one animal model that had implications regarding SPG pathogenesis—this was a study by Safdar and colleagues, 105 in which mice developed hindlimb necrosis when both AT and PC genes were silenced. Also, there remains a widespread misconception that vasopressors are responsible for explaining SPG in the critically ill population; as we have argued elsewhere, 45 46 this seems unlikely, given the key roles of shock, DIC, and natural anticoagulant depletion, as well as the characteristic timing of limb ischemia in relation to proximate onset of shock (consistent with time-dependent decrease in natural anticoagulants) that can be invoked in patients who develop SPG.
